# Nanocrytals-Mediated Oral Drug Delivery: Enhanced Bioavailability of Amiodarone

**DOI:** 10.3390/pharmaceutics14061300

**Published:** 2022-06-18

**Authors:** Anum Munir Awan, Arshad Farid, Shefaat Ullah Shah, Dildar Khan, Fiza Ur Rehman, Muhammad Junaid Dar, Tayyaba Iftikhar, Shakira Ghazanfar, Charis M. Galanakis, Abdulhakeem S. Alamri, Syed Mohammed Basheeruddin Asdaq, Kifayat Ullah Shah

**Affiliations:** 1 Department of Pharmacy, Quaid-i-Azam University, Islamabad 45320, Pakistan; anumawan16@gmail.com (A.M.A.); dildarafridi3@gmail.com (D.K.); drfizaurrehman@gmail.com (F.U.R.); junaiddar@bs.qau.edu.pk (M.J.D.); 2 Gomal Center of Biochemistry and Biotechnology, Gomal University, Dera Ismail Khan 29050, Pakistan; 3 Skin/Regenerative Medicine and Drug Delivery Research, GCPS, Faculty of Pharmacy, Gomal University, Dera Ismail Khan 29050, Pakistan; shefaatbu@gmail.com; 4 Department of Pharmacy, Abdul Wali Khan University, Mardan 23200, Pakistan; t.iftikhar@awkum.edu.pk; 5 Functional Genomics and Bioinformatics, National Agricultural Research Centre, Islamabad 45500, Pakistan; shakira_akmal@yahoo.com; 6 Research & Innovation Department, Galanakis Laboratories, 73131 Chania, Greece; charismgalanakis@gmail.com; 7 Food Waste Recovery Group, ISEKI Food Association, 1190 Vienna, Austria; 8 Department of Biology, College of Science, Taif University, Taif 26571, Saudi Arabia; 9 Department of Clinical Laboratory Sciences, College of Applied Medical Sciences, Taif University, Taif 21944, Saudi Arabia; a.alamri@tu.edu.sa; 10 Centre of Biomedical Sciences Research (CBSR), Deanship of Scientific Research, Taif University, Taif 21944, Saudi Arabia; 11 Department of Pharmacy Practice, College of Pharmacy, AlMaarefa University, Dariyah, Riyadh 13713, Saudi Arabia; sasdag@mcst.edu.sa

**Keywords:** amiodarone HCl, nanocrystals, pluronic F-127, drug delivery, bioavailability enhancement

## Abstract

The aim of this study was to improve the saturation solubility, dissolution profile and oral bioavailability of amiodarone hydrochloride (AMH), a highly lipophilic drug. Stabilizer (Pluronic F-127)-coated AMH nanocrystals (AMH-NCs) were developed by a combination of antisolvent precipitation and homogenization techniques. The optimized formulation comprised pluronic F-127 and AMH at the concentration of 4% and 2% *w*/*v*, respectively. The particle size (PS), zeta potential (ZP) and polydispersity index (PDI) of the optimized formulation was found to be 221 ± 1.2 nm, 35.3 mV and 0.333, respectively. The optimized formulation exhibited a rough surface morphology with particles in colloidal dimensions and a significant reduction in crystallinity of the drug. AMH-NCs showed a marked increase in the saturation solubility as well as rapid dissolution rate when compared with the AMH and marketed product. The stability study displayed that the formulation was stable for 3 months, with no significant change in the PS, ZP and PDI. The in vivo pharmacokinetic study demonstrated the ability of AMH-NCs to significantly (*p* < 0.05) improve the oral bioavailability (2.1-fold) of AMH in comparison with AMH solution, indicating that the production of AMH-NCs using a combination of antisolvent precipitation and homogenization techniques could enhance the bioavailability of the drug.

## 1. Introduction

Low aqueous solubility along with poor bioavailability of new chemical entities are becoming major challenges in the drug delivery, nowadays [1]. The term “low solubility” is applied when the maximum concentration of a drug dissolved in water is <10 mg/mL [2]. Pharmaceutical scientists are considering various techniques, such as, micronization, self-emulsification, salt formation, co-crystallization, cyclodextrin inclusions, solid dispersions, solid lipid nanoparticles and nanocrystallization, to enhance the dissolution and bioavailability of the poorly soluble drugs [3]. Most of these approaches use an excessive number of excipients with a resultant low drug loading capacity, making them suitable only for drugs with a low dose requirement [4,5]. Among various solubility and dissolution enhancing tactics, micronization involves the reduction of the size of the drug particles in the micrometer or nanometer size range [6]. This results in an exponential increase in the interfacial area of contact between the dissolving drug particles and the dissolution medium, thereby enhancing the solubility and dissolution of the drug, with a resultant increase in the bioavailability. Moreover, unlike other techniques, the nanocrystallization approach involves a minimum number of excipients with the additional advantage of high drug loading [4,7]. Currently, more than 50 nanocrystal-based dosage forms have been approved for clinical use [8] and have been formulated in a variety of different dosage forms, including easy to administer ones, such as nanocrystal-based oral film strips [9].

Nanocrystals have drawn attention as an efficient way of delivering poorly soluble drug molecules. As hydrophobic molecules tend to arrange into crystals when exposed to an aqueous medium and form crystals, controlling the crystallization environment can control the shape as well as the size of the drug crystals. In addition, the lattice energy associated with drug molecules provides sufficient stability to the crystals in an aqueous medium. Nanocrystals can, therefore, enhance solubility, improve bioavailability, reduce dose, decrease cost of therapy, and minimize associated side effects. Nanocrystals can be formulated by both top-down as well as bottom-up approaches. Top-down methods involve the breakdown of large particles to the required size range, while bottom-up methods involve building particles from solutions with the addition of antisolvent. The top-down approach comprises high-pressure homogenization and media milling those results in the formation of a nanosuspension, which can be further dried by spray- or freeze drying. The bottom-up approach involves a controlled precipitation by the addition of a drug solution in an antisolvent. Moreover, the selection of a suitable solvent and antisolvent system for the preparation of nanocrystals is extremely critical to yield an optimized formulation, having the desired results [6].

Surfactants function as stabilizers for lipophilic molecules and provide sufficient surface coating to solubilize the poorly soluble molecules. Pluronic F-127 is a non-ionic hydrophilic surfactant which acts as an efficient stabilizer for drug nanocrystals, and it has been used successfully in the development of drug nanocrystals. Amiodarone HCl (AMH) belongs to the BCS class II (low solubility and high intestinal permeability). The logP value of AMH is 6.56, which indicates that it is highly lipophilic in nature [10]. It is a highly effective drug for the treatment of supraventricular and ventricular arrhythmias [11] as well as number of other medical conditions such as Wolff–Parkinson–White syndrome. Following oral administration, AMH has slow and variable absorption due to limited water solubility. Therefore, AMH is the prime candidate for solubility enhancement to improve its bioavailability; hence, reducing its dose, cost of therapy and associated side effects. 

In addition to this, Pluronic F-127, which is implicated as a surfactant for AMH-nanocrystals (AMH-NCs) preparation has an inhibitory effect on the P-glycoprotein, which could help to further improve the bioavailability of AMH [12].

The present study was conducted to enhance the saturation solubility, dissolution profile and oral bioavailability of AMH by producing its nanocrystals. Nanocrystals are known for their ability to enhance drug solubility, improve bioavailability, decrease dose and exhibit high drug loading capacity, along with exceptionally low excipients ratio, thereby reducing toxicity. Therefore, surfactant-coated AMH nanocrystals were developed for solubility enhancement possibly leading to bioavailability enhancement. Pluronic F-127 was selected as the surfactant-coated drug nanocrystals were produced by a combination of antisolvent precipitation and homogenization techniques and then characterized through various in vitro as well as in vivo techniques.

## 2. Materials and Methods

### 2.1. Chemicals and Reagents

AMH was purchased from Sigma Aldrich, Hamburg, Germany. Pluronic F-127, Tween 80 and PEG 6000 were purchased from Kosdac, Merk Schuchardt, Germany. Cardarone (200 mg) tablet and heparin were purchased from a Omega pharmacy, Islamabad Pakistan. Concentrated HCl, disodium hydrogen phosphate, dipotassium hydrogen phosphate and methanol were purchased from BDH laboratory supplies, England. Size 0 capsule and avecil-200 was gifted by Wise pharmaceuticals, Pakistan. The rest of the chemicals used were of pure analytical grade.

### 2.2. Methods

#### 2.2.1. Preparation of AMH Nanocrystals

The AMH-NCs were prepared through an antisolvent precipitation method followed by high shear homogenization to obtain NCs of the required size. To prepare the solvent phase, AMH (2% of total formulation) was first dissolved in a suitable quantity of methanol. The prepared solvent phase was added dropwise into the antisolvent phase (ratio of solvent to antisolvent 1:10) containing pre-screened surfactant (4% of total concentration) with a stirring speed of 1200 rpm at room temperature. The formulation was subjected to a rotary evaporator for 20 min at 100 rpm and 70 °C to evaporate the organic phase. This suspension was then subjected to a homogenizer at a speed of 7000 rpm for 20 min. The developed formulation was further freeze-dried to obtain AMH-NCs dried powder [1].

#### 2.2.2. Optimization Parameters

A hit and trial method was utilized for optimization of AMH-NCs. Factors that were taken into consideration for the purpose of optimization (screened from pre-formulation studies) were selection of suitable surfactant, stirring speed, stirring time, solvent–antisolvent ratio, homogenization speed and time. A total of 12 trials were generated by varying the surfactant (Pluronic F-127, Tween 80 and PEG 6000) and their concentrations ranging from 2 to 5%. After selection of a suitable surfactant and its concentration, other optimization parameters were evaluated in terms of particle size and polydispersity index.

To examine the effect of solvent–antisolvent ratio on the particle size, AMH-NCs were prepared in different solvent–antisolvent ratios (1:5, 1:10 and 1:15). The impact of stirring speed and stirring time on the aforementioned responses were evaluated. Along with that, the effect of drug concentration (2–4%) on drug loading was also estimated. Likewise, the effect of homogenization speed was also taken into consideration. 

#### 2.2.3. Characterization of Optimized AMH Nanocrystal Formulation

##### Drug Content Analysis

To determine the AMH content in the nanocrystal formulation, the prepared nanosuspension was centrifuged at 20,000 rpm for 45 min (at 4 °C). The supernatant was then analyzed for the drug content through a UV-Visible double beam spectrophotometer (Halo DB-20, Dynamica, Victoria, Australia).

##### Particle Size Distribution, Zeta Potential and Polydispersity Index

The prepared NCs were assessed for particle size, polydispersity index (PDI) and zeta potential (ZP). A sample for zeta analysis was prepared by taking 10 µL of sample solution in an Eppendorf tube and diluting it with 1 mL of distilled water for suitable scattering intensity. A Zetasizer (Malvern Instrument; Worcestershire, UK) was used to determine the mean particle size (z-average) and PDI, which is regarded as a measurement of the width of size distribution and ZP. Each sample was analyzed in triplicate with 13 runs and 60 s duration in each measurement at a temperature of 25 °C.

##### Fourier Transformer Infrared Spectroscopy (FTIR)

The interaction of the drug with other polymers was studied using the Fourier transform infrared spectrometer (Perkin-Elmer spectrum 100). This test was performed to check any interaction among the drug and excipients used in the formulation.

##### Scanning Electron Microscopy

The particle morphology of the nanocrystal formulation was visualized using a scanning electron microscope (SEM, JEOL JSM-6490A, microscope Japan) at an excitation voltage of 10 keV. First, the samples were mounted on double-sided carbon sticky tabs and further sputtered with gold (10 nm thickness) before imaging.

##### X-ray Powder Diffraction

The X-ray powder diffraction (XRPD) analysis of pure AMH and optimized AMH-NCs formulation was conducted using a Bruker D8 X-ray powder diffractometer (STOE, Karlsruhe, Germany) with Cu-Kα radiation (2 Å). Approximately 150 mg samples were gently consolidated in the natural sample holder and scanned at 40 kV and 40 mA from 3° to 40° 2θ with a scan speed of 1 s at an increment rate of 0.01 s.

#### 2.2.4. In Vitro Characterization

##### Saturation Solubility

Saturation solubility studies were performed using a shaker bath (Memmert SV-1422, Hamburg, Germany) and the temperature was maintained at 37 ± 0.5 °C. Pure drug, physical mixture of pure AMH with selected surfactant, and optimized formulation were used. The pure drug, physical mixture and formulation equivalent to 5 mg of drug were added separately into water and a phosphate buffer (pH 6.8). A sample of 500 μL was collected after 48 h and subjected to ultracentrifugation at 20,000 rpm at 4 °C for 15 min. The supernatant was suitably diluted with distilled water and analyzed through the UV-Visible double beam spectrophotometer for determination of saturation solubility of AMH.

##### In Vitro Dissolution

Dissolution tests were performed using the USP dissolution apparatus II (Tianjin Tianda Tianfa Technology Co., Ltd., Tianjin, China) in 900 mL buffers of pH 6.8, pH 1.2 and distilled water at 37 ± 0.5 °C. The paddle speed was set at 100 rpm. The AMH powder, marketed product (cardarone 200 mg tablet) and AMH-NCs (equivalent to 200 mg of AMH) were filled into hard gelatin capsules (size 0), which were then placed in sinkers and placed in the dissolution apparatus. Samples of 5 mL were then withdrawn at predetermined time intervals (1, 5, 15, 20, 30, 45 and 60 min), and replaced with an equivalent amount of fresh medium to maintain the sink conditions. The withdrawn samples were all passed through 0.22 µm filters, and the filtrates were subjected to quantitative analysis using the UV-Visible double beam spectrophotometer at 237 nm. The experiments were carried out in triplicate.

#### 2.2.5. In-Vivo Characterization

##### In Vivo Pharmacokinetics

Male Sprague Dawley SD rats having a body weight ranging from 260 to 320 g were used for all in vivo experiments. The in vivo studies were carried out in accordance with the guidelines evaluated and approved by the ethical committee of Quaid-i-Azam University, Islamabad, Pakistan. A total of 12 rats were randomly divided into 3 groups (n = 4)—group I: saline-treated group, group II: treated with pure AMH and group III: treated with AMH-NCs. Rats were fasted overnight before the experiment but given free access to water. A dose equivalent to 3 mg/kg body weight was administered orally utilizing an oral gavage tube. The suspension was prepared with distilled water: the dosing concentration was 0.5 mg-AMH/mL. Blood samples were collected from the lateral tail vein at 0, 0.5, 1, 2, 3, 5, 7, 9, 18, 24 and 48 h after oral administration. Each blood sample was centrifuged at 5000 rpm for 10 min to prepare plasma samples. Plasma AMH concentrations were determined through HPLC. Briefly, the plasma (0.1 mL) samples were mixed with 20 µL of internal standard solution. Then, 1.5 ml of acetonitrile was added to the plasma and mixed for 5 min. The mixture was centrifuged at 11,000 rpm for 5 min, and the supernatant was transferred and dried under nitrogen flux. The dried residue was mixed with 0.4 ml of methanol and then 20 µL of the sample was injected into the column. The mobile phase was 2% triethylamine solution (adjusted to pH 4.0 with phosphoric acid): methanol (18:82 *v*/*v*), which was delivered at an isocratic flow rate of 1.0 mL/min at 40 °C, and relative humidity (RH) was less than 50% [13]. The concentration data for AMH and AMH-NCs were dose-normalized and plotted as a drug concentration–time curve. The area under the curve (AUC) was calculated using the trapezoidal rule [1,13,14].

#### 2.2.6. Storage Stability

Optimized AMH-NCs were stored in a sealed glass vial and kept inside a stability chamber at temperature of 25 °C for 3 months to assess their stability. After 3 months, the formulation was evaluated for particle size distribution, zeta potential and polydispersity index.

### 2.3. Statistical Analysis

All the results are exhibited as mean ±standard deviation (SD). Data were analyzed using Student’s *t*-test or one-way analysis of variance (ANOVA) (GraphPad Prism software Inc., San Diego, CA, USA); *p* values < 0.05 were considered as statistically significant.

## 3. Results and Discussion

### 3.1. Optimization of Nanocrytals

In order to achieve a smaller particle size and a narrow distribution of AMH-NCs, an appropriate stabilizer, stabilizer concentration, stirring speed, homogenization time, homogenization speed and injection rate is required. In this study, pure stabilizers (Pluronic F-127, PEG 6000 and Tween 80) were being utilized to prepare AMH-NCs. A varied concentration of different stabilizers was evaluated. Based on Zetasizer results of the particle size and PDI, Pluronic F-127 at the concentration of 4% was selected to prepare the AMH-NCs.

#### 3.1.1. Formulation, Characterization and Optimization of AMH-NCs on the Basis of Zetasizer Results

The AMH-NCs were prepared by the antisolvent precipitation method. In this method, the injection of drug solution (solvent phase) to the aqueous phase (antisolvent phase) generates a high supersaturation. This supersaturation leads to a rapid nucleation rate and produces a substantial number of nuclei, which in turn reduces the solute mass for the subsequent crystal growth. Growth of these nucleating crystals are arrested by the surface-active agents through the mechanism of stearic or electrostatic hindrance. Water is the widely used antisolvent for most of the hydrophobic drugs. Solvent must be chosen depending upon the solubility of drug, i.e., in which the drug is solubilized properly. However, the stabilizer must exhibit a good affinity to the drug and must possess a fast rate of diffusion as well as an effective adsorption to the drug molecules in the solvent antisolvent mixture [15]. Therefore, the mixture of solvent and stabilizer is crucial to the nanocrystal production. In this experimental study, three different pure surfactants such as PEG 6000, Pluronic F-127, Tween 80 were evaluated at four different concentration ranges (2%, 3%, 4%, 5%). The results are shown in Table 1. 

Three different surfactants were opted for the stabilization of AMH-NCs obtained by the antisolvent precipitation method. As a general trend, it was observed that as the concentration of surfactant was incremented, the particle size and the PDI were reduced. In other words, the small-sized and stabilized AMH-NCs were obtained. According to the particle size and PDI results stated in Table 1, 4% Pluronic F-127 was found to be the most appropriate surfactant owing to superior stabilization of AMH-NCs. With 4% Pluronic F-127, the particle size was found to be 311 ± 2.43 nm and 221.01 ± 1.2 nm without and with homogenization, respectively. Moreover, it also stabilizes the AMH-NCs to a greater extent as evident by the PDI of 0.133 ± 0.008. By further increase in Pluronic F-127 concentration, the larger-sized nanocrystals with PDI of 0.154 ± 0.017 were attained. The pretext of this distinguished observation was that at higher concentrations of Pluronic F-127, instead of drug adsorption, it transform into the micelles. These micelles competed for drug surface adsorption, and the total adsorption at the interface began to decrease as the micelles became more numerous [16,17]. Thus, a higher surfactant concentration (in which micelles began to form) could mean less surfactant adsorption, which would lead to destabilization of AMH-NCs. However, PEG 6000 failed to stabilize AMH-NCs. Chemically, polyethylene hydroxide is a linear hydrophilic homopolymer. To stabilize AMH-NCs, interaction between the drug and the stabilizer, such as hydrophobic interactions, ionic bond and hydrogen bonds are mainly required, which results in the adsorption of the stabilizer on the drug surfaces. PEG lacks hydrophobic moieties and other functional groups, which is the major problem in adsorption on the drug surface [18]. Moreover, the results with Tween 80 were also encouraging; however, owing to small particle size and superior stability, 4% Pluronic F-127-based nanocrystals were optimized and subjected to further characterization as well as in vivo studies. 

#### 3.1.2. Effect of the Homogenization on the Particle Size of Nanocrystals

The high-speed homogenization was found to have a significant effect on the particle size and PDI of the designed nanocrystals. The marked decrement in both erstwhile-stated factors was observed. These results can clearly be observed from the data mentioned in Table 2. The shear forces of the high-pressure homogenization produced the small-sized nanocrystals compared to the nanocrystals obtained from simple antisolvent precipitation method (without homogenization). Regarding the formulation F-3, the homogenization significantly minimized the particle size to 221 ± 1.2 nm, as shown in Table 1.

### 3.2. Characterization of Nanocrystals

#### 3.2.1. Particle Size Distribution, Polydispersity Index and Zeta Potential

**Figure 1:**
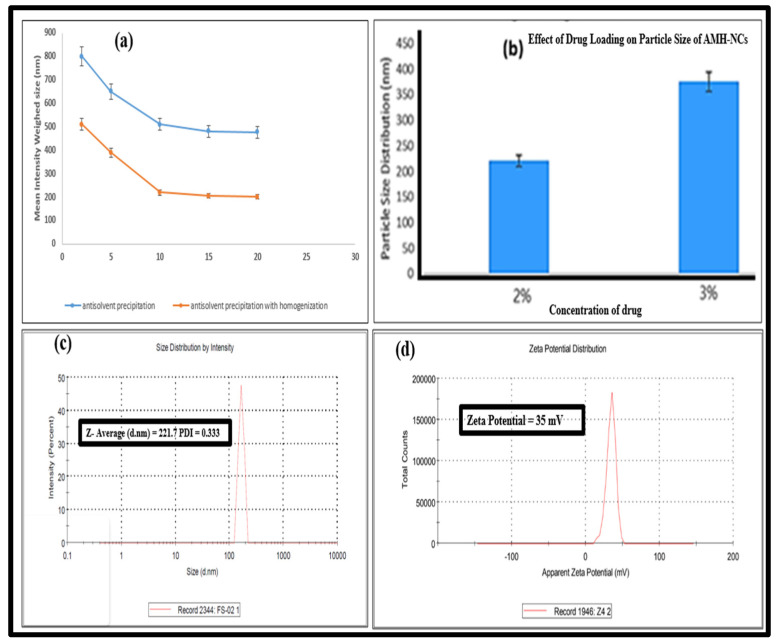
(**a**) Mean intensity weighed size of AMH-NCs prepared by various volume ratios for antisolvent to solvent. (**b**) Effect of drug loading on particle size distribution (nm) of AMH-NCs. (**c**) Particle size, polydispersity of AMH-NCs and (**d**) zeta potential of AMH-NCs.

Optimized AMH-NCs were subjected to the particle size, polydispersity and zeta potential measurements. The mean value for the particle size, PDI and ZP values of AMH-NCs were 221 ± 1.2 nm, 0.333 and 35.3 mV, respectively (Figure 1c,d). Typically, the value for surface charges arises from the ionization of the particle surfaces, or adsorption of surfactants onto the surface [19,20]. In the present study, the value of zeta potential is 35.3 mV, which would be beneficial for the stability of AMH-NCs.

#### 3.2.2. Fourier Transformed Infrared (FTIR) Spectroscopic Analysis 

**Figure 2:**
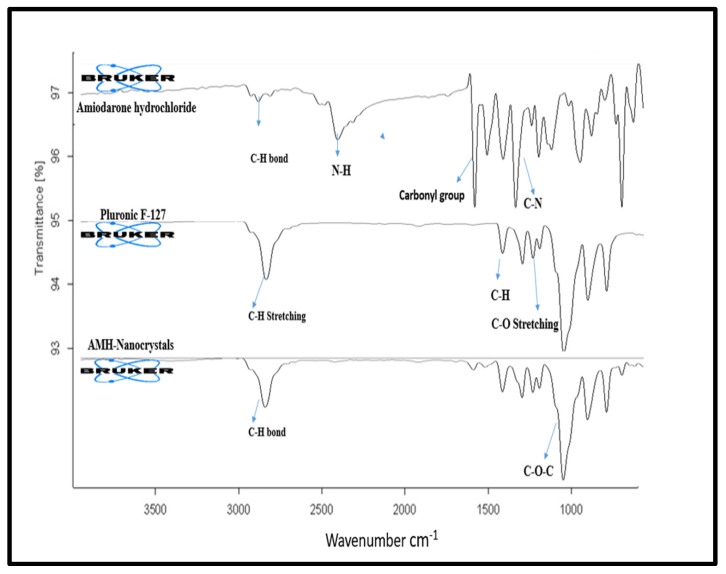
Representing the FTIR spectrum of pure amiodarone (AMH), Pluronic F-127 and AMH-NCs.

Pure AMH and precipitated AMH-NCs showed peaks at wavelengths of 2800 and 3250 cm^−1^ due to the stretching vibration of the aromatic and aliphatic -CH bond. The band at 2476 cm^−1^ is produced due to the stretching vibration of -NH. The carbonyl group of diiodophenylketone moiety of compound produced a strong absorption band at 1635 cm^−1^. Stretching of the -CN bond produced a strong band at 1375 cm^−1^. The strong absorption band at 1249 cm^−1^ is attributed to ether group stretching. The band at 1026 cm^−1^ is attributed to the furan ring breathing. Aromatic -CC ring quadrant stretching at 1557 and 1530 cm^−1^. Moreover, it shows ketonic -CO bending at 1284 cm^−1^ (Figure 2) [21].

The FTIR spectrum of Pluronic F-127 showed characteristic peaks at 3530, 2890, 1402, and 1110 cm^−1^ corresponding to asymmetric stretching vibration of -OH, -CH, -CC, -CO, respectively. The absorption band at 1469 cm^−1^ is attributed to -CH group bending vibration. The characteristic peaks at 1345, 1242, 1281, 1148, 1113 and 1061 cm^−1^ indicate -CO stretching vibration (Figure 2) [1].

AMH-NCs with Pluronic F-127 as a stabilizer exhibit same IR spectrum so the chemical structure did not change by solvent–antisolvent precipitation method. FTIR analysis was done to point out any interaction between the drug and surfactant molecules. There was no chemical interaction observed between AMH and Pluronic F-127 except overlapping of band between 2880 and 2980 cm^−1^, indicating the stretching vibration for both the AMH and the Pluronic F-127 as shown in Figure 2.

#### 3.2.3. Morphological Analysis

**Figure 3:**
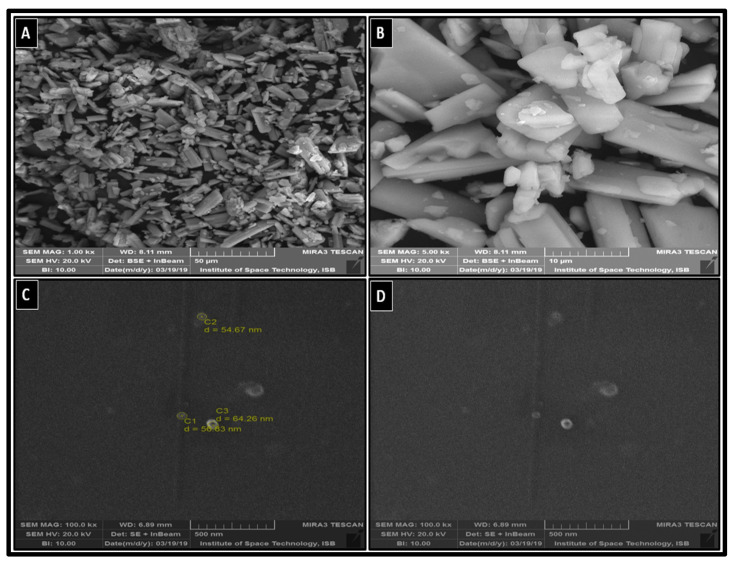
(**A**) SEM images of pure amiodarone (AMH) 50 micrometer and (**B**) 10 micrometer resolution while the (**C**,**D**), representing the SEM images of AMH-NCs with 500 nm resolution.

The morphological analysis of pure AMH (Figure 3A,B) and AMH-NCs (Figure 3C,D) was executed using a scanning electron microscope (SEM). The results showed that the surface morphology of both AMH and AMH-NCs is same, but the particle size is considerably reduced in the AMH-NCs.

#### 3.2.4. Characterization by X-ray Powder Diffraction (XPRD)

**Figure 4:**
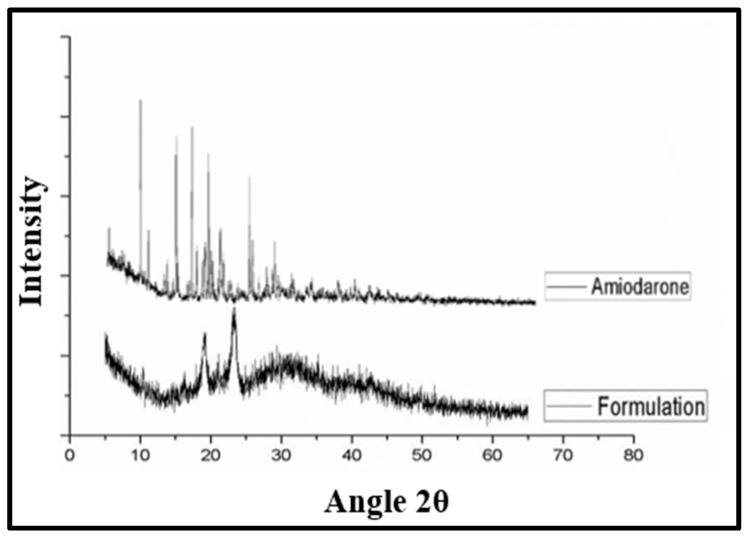
XRPD pattern of pure amiodarone (AMH) and formulation (AMH-NCs).

Solubility, rate of dissolution and bioavailability are the properties that depend upon the solid-state parameter of the drug particles. The XPRD pattern of the pure AMH and AMH-NCs is shown in Figure 4. The XPRD peaks of AMH showed intense peaks for the drug at 2Ɵ equal to 7.3, 13.5, 15.4, 21.1, 21.3 and 25.7° (Figure 4) [21]. Whereas, the intensity of AMH has decreased and some peaks are suppressed in AMH-NCs, thus indicating the reduction in drug crystallinity.

### 3.3. Saturation Solubility

**Figure 5:**
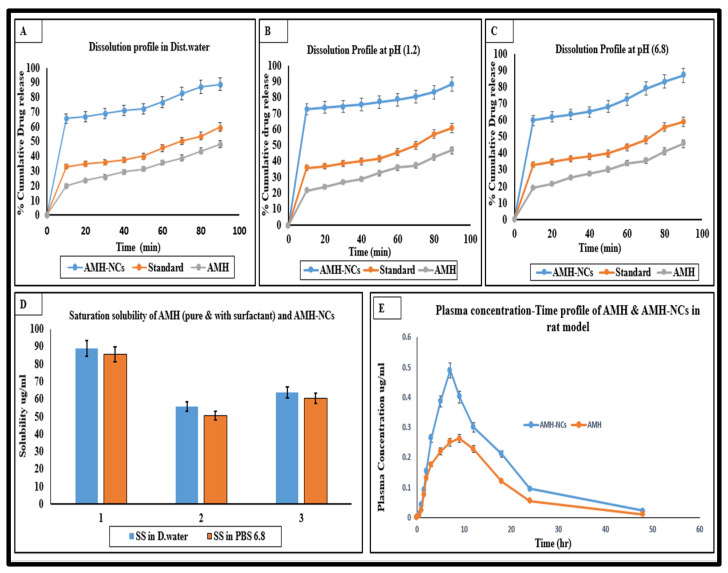
(**A**–**C**) Dissolution profile in distilled water pH 1.2 and pH 6.8. (**D**) Saturation solubility (SS) of AMH, Pluronic F-127 and AMH-NCs in distilled water and PBS (pH 6.8). (**E**) Plasma drug concentration–time curve of AMH in rats after oral administration of AMH suspension and optimized AMH-NCs formulations equivalent to 3 mg/kg of AMH (n = 4; mean ± SD).

The saturation solubility of the AMH-NCs, pure drug and physical mixture of AMH and Pluronic F-127 was investigated. The saturation solubility of AMH-NCs was found to be 88.9 and 86 µg/mL, pure AMH has the saturation solubility of 55.3 and 50.0 µg/mL, and the mixture of AMH with Pluronic F-127 showed saturation solubility of 63.7 and 61.5 µg/mL in water and phosphate buffered saline (PBS) of pH 6.8, respectively (Figure 5). The saturation solubility of the optimized nanosuspension formulation showed a significant increase in the solubility as compared with the pure AMH powder. It is reported elsewhere that a significant increase in solubility due to increased curvature of drug nanoparticles is not expected with drug nanosizing; rather, rapid dissolution kinetics is expected to be significantly increased with particle size reduction. The increase in saturation solubility of AMH-NCs can be explained according to the Ostwald–Freundlich equation:

(1) $$S=S_{\infty }.\;\exp\;\left[\frac{2ɣM}{r\rho RT}\right]$$

where S is the saturation solubility of the AMH-NCs, S_∞_ is the saturation solubility of the infinitely large crystals, ɣ is the crystal medium interfacial tension, *M* is compound molecular weight, *r* is the particle radius, *ρ* is the density, *R* is the gas constant and *T* is the temperature. A decrease in *r* certainly causes an increase in saturation solubility of drug [18].

### 3.4. Dissolution Test

The lyophilized AMH-NCs showed a significant (*p* < 0.05) increase in the rate and extent of dissolution, and the rate of dissolution was maintained at a higher level throughout the experiment as compared to the pure drug and marketed product (Cardarone tablet). Within 10 min, almost 65.4% of AMH-NCs was dissolved in the distilled water, while only 33% and 20% of the marketed product and pure AMH powder were dissolved, respectively. After 90 min of dissolution, 88.77% of drug was released for the AMH-NCs compared to 48 and 59.6% from the pure AMH powder and marketed product, respectively. Similarly, dissolution profile in PBS (pH 6.8) and simulated gastric fluid (pH 1.2) also indicated a significant increase in the dissolution efficiency of AMH-NCs as compared with pure AMH and marketed product (Figure 5A–C).

No agglomeration of drug particles was observed during dissolution, this may be due to the presence of stabilizer in the formulation of AMH-NCs. The Noyes–Whitney equation describes the exponential increase in the surface area of drug molecules, which indicates that higher surface to volume ratio leads to hydration of molecules, along with that particle size reduction causes reduction in the thickness of diffusion layer [22]. Hence, nanometer size range particles are the main cause for the improved dissolution profile of the drug product. Hydration of drug particles causes better dispersion, which also leads to improved dissolution. Reduction in the crystallinity, improved dissolution and reduction in the diffusion layer thickness are the potential reasons behind enhanced bioavailability.

### 3.5. In Vivo Studies

#### In Vivo Pharmacokinetics Studies

The plasma concentration–time profiles and the main pharmacokinetic parameters after oral administration of pure AMH suspension and AMH-NCs in male SD rats are presented in Figure 5C. As expected, the oral absorption of pure AMH suspension was found to be very low due to its pure dissolution properties. A significant increase in the oral absorption was observed with AMH-NCs as shown in Table 3. The enhanced dissolution profile of AMH-NCs could be the possible reason behind it. Moreover, the direct uptake of the intact nanoparticles by the Peyer’s patches of the GI lymphoid tissue might be another possible reason for the improved absorption of the AMH-NCs. This approach may provide a route to avoid first-pass metabolism of AMH [23,24]. The plasma concentration in rats treated with AMH-NCs was 1.6-fold higher than in rats treated with pure AMH. The time to reach Cmax AMH-NCs was also slightly lower than that of pure AMH suspension.

### 3.6. Storage Stability of Optimized AMH-NCs Formulation

The performance parameter such as Physical appearance, particle size (ZP) and drug content of the optimized formulation of AMH-NCs following storage at 25 °C for 3 months is shown in Table 4. The data showed that AMH-NCs are stable for said time.

## 4. Conclusions

Nanosizing presents a potential approach for enhancement in solubility of hydrophobic drug moieties. The present research investigated the potential of combination of size-reduction techniques such as antisolvent precipitation and homogenization for the development of AMH-NCs. The formulation was extensively optimized utilizing three different surfactants (Pluronic F-127, Tween 80 and PEG 6000) and found to possess the adequate particle size and extremely homogenous particle size distribution. The AMH-NCs were found to be stable for 3 months in terms of particle size, PDI and zeta potential. In addition, in vivo pharmacokinetics in Sprague Dawley rats showed remarkable improvement in oral bioavailability of AMH-NCs.

## Figures and Tables

**Table 1:** Effect of stabilizer on the particle size and stability of AMH-NCs.

Formulation Code	Drug (%age)	Pluronic F-127 (%)	Tween 80 (%)	PEG 6000 (%)	Antisolvent Precipitation		Antisolvent Precipitation with Homogenization	
					Size (nm)	PDI	Size (nm)	PDI
F1	2	2	-	-	581 ± 2.1	0.438	601 ± 2.1	0.41
F2	2	3	-	-	483 ± 1.7	0.211	392 ± 1.8	0.342
F3	2	4	-	-	311 ± 2.43	0.344	221 ± 1.2	0.333
F4	2	5	-	-	319 ± 1.8	0.296	312 ± 1.6	0.291
F5	2	-	2	-	586 ± 2.4	0.821	473 ± 2.1	0.687
F6	2	-	3	-	402 ± 1.9	0.568	448 ± 1.8	0.553
F7	2	-	4	-	433 ± 2.3	0.399	364 ± 2.1	0.200
F8	2	-	5	-	400 ± 1.9	0.378	401 ± 1.9	0.234
F9	2	-	-	2	695 ± 2.4	0.761	540 ± 2.4	0.431
F10	2	-	-	3	542 ± 1.9	0.654	531 ± 1.8	0.419
F11	2	-	-	4	490 ± 2.0	0.543	463 ± 1.9	0.365
F12	2	-	-	5	489 ± 2.1	0.540	471 ± 1.9	0.433

**Table 2:** Particle size distribution and polydispersity index of AMH using different concentrations of surfactants (Pluronic F-127, PEG 6000 and Tween 80) by antisolvent precipitation method with and without homogenization.

Method	Parameters	Formulation Code (Surfactant Concentration Used)											
		Pluronic F-127 2%	Pluronic F-127 3%	Pluronic F-127 4%	Pluronic F-125 5%	PEG 6000 2%	PEG 6000 3%	PEG 6000 4%	PEG 6000 5%	Tween 80 2%	Tween 80 3%	Tween 80 4%	Tween 80 5%
Sol-precipitation without homogenization	PS (nm)	561 ± 2.1	483 ± 1.7	311 ± 2.43	319 ± 1.8	695 ± 2.4	542 ± 1.9	490 ± 2.0	489 ± 2.1	586 ± 2.4	402 ± 1.9	433 ± 2.3	400 ± 1.9
	PDI	0.438	0.211	0.344	0.296	0.761	0.654	0.543	0.540	0.821	0.568	0.399	0.378
Sol- precipitation with homogenization	PS (nm)	601 ± 2.1	392 ± 1.8	221 ± 1.2	312 ± 1.6	540 ± 2.4	531 ± 1.8	463 ± 1.9	471 ± 1.9	473 ± 2.1	448 ± 1.8	364 ± 2.1	401 ± 1.9
	PDI	0.41	0.342	0.207	0.291	0.431	0.419	0.365	0.433	0.687	0.553	0.200	0.234

**Table 3:** Pharmacokinetic parameters of pure AMH and AMH-NCs after single oral dose (3 mg/kg) to SD rats. *p* < 0.05 considered significant when compared to AMH.

Formulation	Test		
Parameters	Cmax (ng/mL)	Tmax (hr)	AUC_0–48_ (ng/mL)
AMH-NCs	0.491 ± 0.045	6.78 ± 0.24	7387 ± 12.41
AMH	0.299 ± 0.037	7.12 ± 0.27	5900 ± 9.83

**Table 4:** Stability studies at 0,1 and 3 months’ time period at 25 °C and RH < 40%.

Time Month	Physical Appearance	Color Change	Particle Size	Drug Content
0 month	Homogenous	No change(white)	221 ± 0.14	92.54 ± 0.45
1 month	Homogenous	No change	223 ± 1.29	90.91 ± 1.22
3 month	Homogenous	No change	227 ± 1.31	90.87 ± 0.51

## Data Availability

The data are included in the manuscript.
